# Do allergic dogs exhibit features of a severe steroid-resistant allergic airway phenotype?

**DOI:** 10.1186/1476-9255-10-S1-P40

**Published:** 2013-08-14

**Authors:** Edward G Barrett, Christopher M Royer, Karin Rudolph, Phillip J Kuehl

**Affiliations:** 1 Lovelace Respiratory Research Institute, Albuquerque, NM, 87108, USA

## Background

Most asthmatic patients respond favorably to corticosteroid therapy, however, a subpopulation of patients (5-10%) display persistent immune activation and airway inflammation, despite high doses of oral/inhaled corticosteroids. The development of more predictive animal models that incorporate the severe steroid-resistant phenotype are needed to better define translational biomarkers and therapeutic targets for this patient population.

## Materials and methods

For the last 20 + years LRRI has been breeding and sensitizing Beagle dogs such that they exhibit multiple allergic phenotypes (asthma, dermatitis, and, rhinitis). Over those years higher doses of steroids seem to be required to see a similar effect. In three recent studies, dogs (n=6-8/study) previously sensitized to ragweed (RW) allergen were pretreated with vehicle or a) 1 week oral prednisolone (15 mg/day), b) 2 weeks oral prednisolone (15 mg/day), or c) 2 weeks inhaled fluticasone propionate (350 μg lung deposited dose; BID). Blood leukocytes and lung inflammatory (bronchoalveolar lavage; BAL) cells were measured before during and after treatment-allergen challenge. Changes in lung function (airway resistance and compliance) were measured immediately after RW challenge and at 24 hours post RW (MCh dose response for airways hyperreactivity).

## Results

One week of prednisolone treatment did not lead to any significant attenuation of pulmonary function endpoints or lung inflammation (except for a reduction in BAL lymphocytes). Two weeks of prednisolone treatment led to a significant reduction in BAL cells (neutrophils, eosinophils, and lymphocytes) and in blood eosinophils, however this did not correlate with any significant change in airway function. Two weeks of inhaled fluticasone led to a significant reduction in BAL cells (neutrophils, eosinophils, and lymphocytes) and in blood eosinophils, however this did not correlate with any significant change in airway function. Age of the dogs (e.g. number of allergen challenges over the years) or their level of responsiveness to allergen challenge did not seem to predict a better or worse response to steroids.

## Conclusions

Dogs sensitized to RW exhibit some features of a severe steroid-resistant phenotype: high doses of oral or inhaled steroid required to see an effect on inflammation, with no significant effects on airway function. However, it raises the question as to how one defines steroid resistance in an animal model where the animals underlying allergic disease is clinically stable unless purposely challenged with allergen and then only receive steroid treatment as a positive control during a treatment study.

